# Improvement of light penetration in biological tissue using an ultrasound-induced heating tunnel

**DOI:** 10.1038/s41598-020-73878-4

**Published:** 2020-10-15

**Authors:** Zong-Han Hsieh, Ching-Hsiang Fan, Yi-Ju Ho, Meng-Lin Li, Chih-Kuang Yeh

**Affiliations:** 1 Department of Biomedical Engineering and Environmental Sciences, National Tsing Hua University, Hsinchu, Taiwan; 2 Department of Biomedical Engineering, National Cheng Kung University, Tainan, Taiwan; 3 Department of Electrical Engineering, National Tsing Hua University, Hsinchu, Taiwan; 4 Institute of Photonics Technologies, National Tsing Hua University, Hsinchu, Taiwan

**Keywords:** Biological techniques, Optics and photonics

## Abstract

The major obstacles of optical imaging and photothermal therapy in biomedical applications is the strong scattering of light within biological tissues resulting in light defocusing and limited penetration. In this study, we propose high intensity focused ultrasound (HIFU)-induced heating tunnel to reduce the photon scattering. To verify our idea, Monte Carlo simulation and intralipid-phantom experiments were conducted. The results show that the thermal effect created by HIFU could improve the light fluence at the targeted region by 3% in both simulation and phantom experiments. Owing to the fluence increase, similar results can also be found in the photoacoustic experiments. In conclusion, our proposed method shows a noninvasive way to increase the light delivery efficiency in turbid medium. It is expected that our finding has a potential for improving the focal light delivery in photoacoustic imaging and photothermal therapy.

## Introduction

The advent of photonic technology has played a critical role in novel biological discoveries and clinical applications, such as biomedical imaging and optical manipulation, stimulation, and therapy. Applications involving light beams illuminating biological media are greatly affected by intratissue propagation, since desired goals can be impossible to achieve when there is insufficient light energy or poor spatial resolution. For example, the poor delivery of light energy in photoacoustic imaging will decrease the photoacoustic signals and the imaging contrast^1–3^. Optical scattering is the most important obstacle because this rapidly decreases the light intensity and widens the light beam during propagation^4^. The energy loss from scattering can be reduced by inserting an optical fiber into the target region, but this can potentially cause invasive damage^5^. It is clear that the development of a new approach for noninvasively reducing light scattering in biological media would fundamentally increase its penetration ability and treatment efficiency, and also reduce off-target effects.

The existing methods for decreasing scattering can typically be divided into two types: wavefront shaping^6–8^ and optical clearing^9–11^. In a wavefront shaping process, ultrasound could be used as a guide to modulate and refocus light. However, the requirement for a perpendicular spatial arrangement between the light and ultrasound transducers and the time-consuming calculations means that these methods are not suitable for real-time in vivo applications. In an optical-clearing technique, a chemical optical clearing agent is used to lower the scattering coefficient in tissue and thereby improve the light penetration. However, the use of chemical agents may cause tissue injury (e.g., dehydration), making them only appropriate for postmortem applications. Based on the same theory of using an optical clearing agent, some researchers have proposed optical clearing methods based on mechanical forces (i.e., pressure) to alter the optical properties of tissues^12–14^. For example, Chamanzar et al. found that ultrasound could increase the penetration of light by temporally changing the optical refractive index of tissue^14^. Moreover, because no chemical agent was involved, the change was reversible. Unfortunately, their backward-mode system based on the transmission of ultrasound standing waves was not suitable for in vivo applications. An ideal system for improving light penetration within tissue for in vivo applications must conform to the following criteria: noninvasive, high temporal resolution, localization, simple setup, and tissue safety.

**Figure 1:**
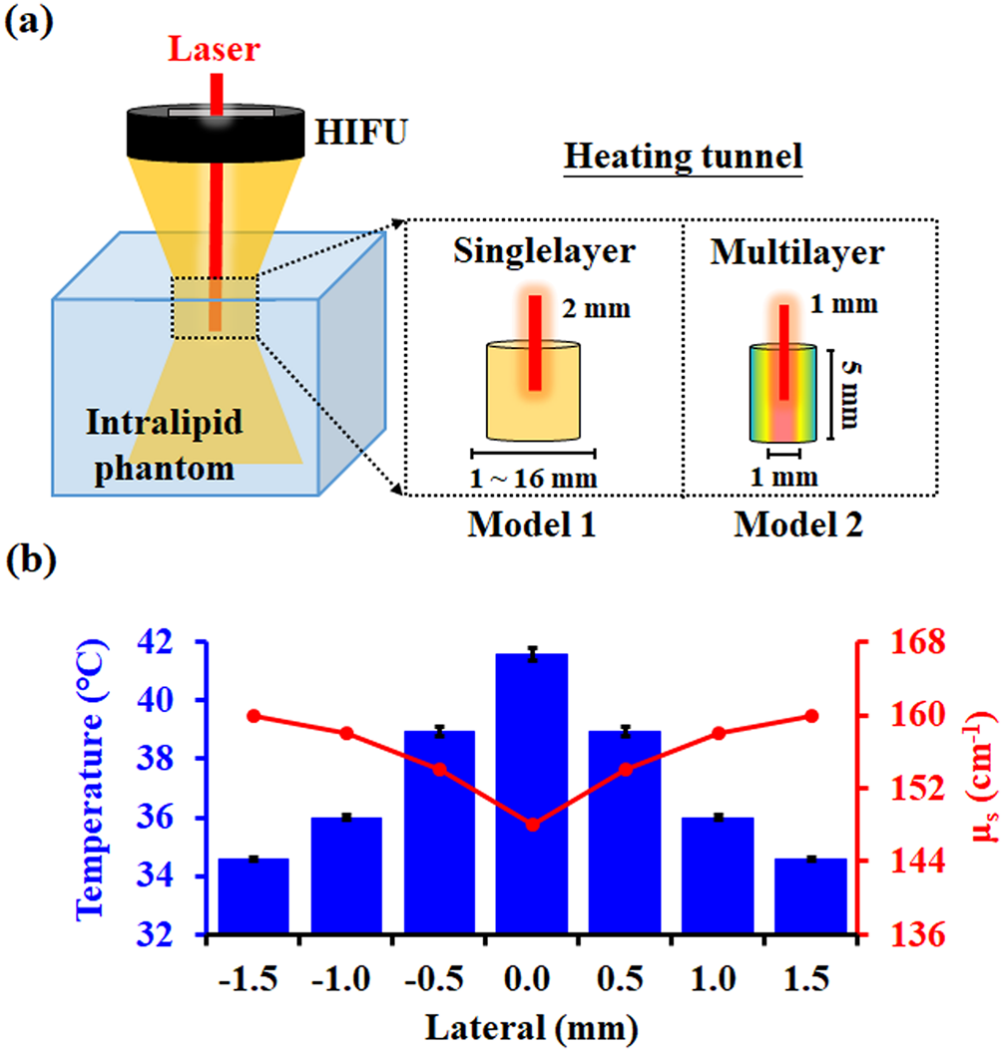
(**a**) Illustration of the two simulation models. Model 1: optimizing the size and the temperature range of the heating tunnel. Model 2: simulating the heating tunnel produced by HIFU within the intralipid phantom. (**b**) Temperature and scattering-coefficient distributions of the four-layer heating tunnel used in model 2.

**Figure 2:**
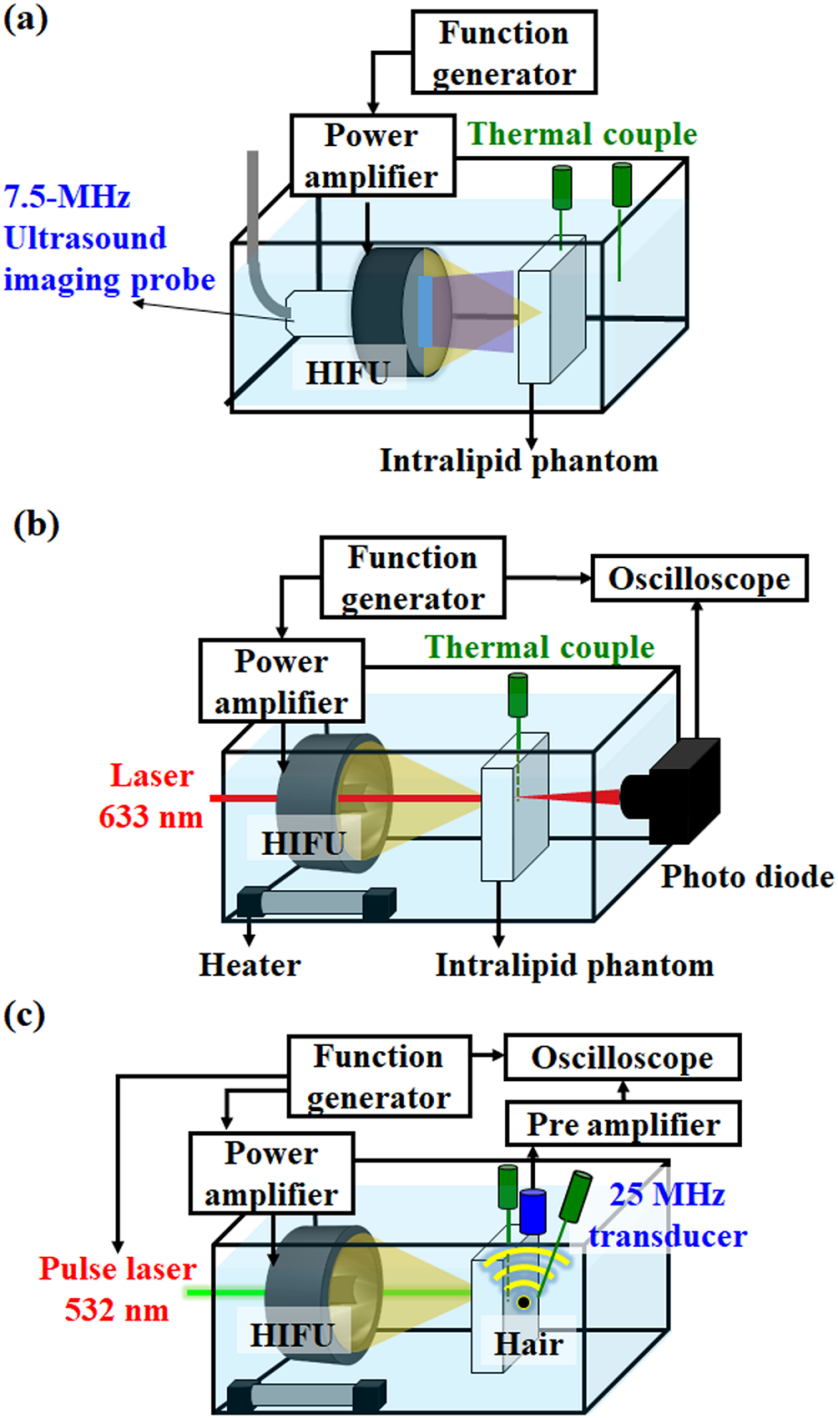
(**a**) Experimental setup to detect the inertial cavitation effect and record the temperature change. (**b**) Experimental setup to verify that the optical scattering could be reduced by the HIFU-induced heating tunnel. (**c**) Photoacoustic experimental setup for improving photoacoustic signals by the HIFU-induced heating tunnel.

Our strategy focused on the concept of heat-mediated changes in tissue scattering coefficients^15,16^. A medium expands when the temperature increases, which reduces light scattering. Moreover, cell membranes are mainly formed by phospholipids, and their fluidity would increase when the temperature increases from 25 to 45 °C due to undergoing a phase transition^17^. Although this potential mechanism of temperature-dependent scattering reduction has been demonstrated previously, there are very few reports of this approach being applied in optical-related techniques. In this study, we aimed to develop a safe noninvasive method for improving the delivery of light energy by using high-intensity focused ultrasound (HIFU) as a heating source to create a heating tunnel in the scattering medium. HIFU was suitable for this study because it has been widely used to achieve noninvasive and high-accuracy thermal ablation in clinical applications^18^. The HIFU enhancement of light delivery was first simulated in Monte Carlo simulations, and the performance of the technique was validated in an intralipid phantom. Finally, an HIFU heating system was integrated with a photoacoustic system to verify the improvement of photoacoustic signals resulting from the improved light delivery (Figs. 1, 2).

## Results

### Monte Carlo simulation results

**Figure 3:**
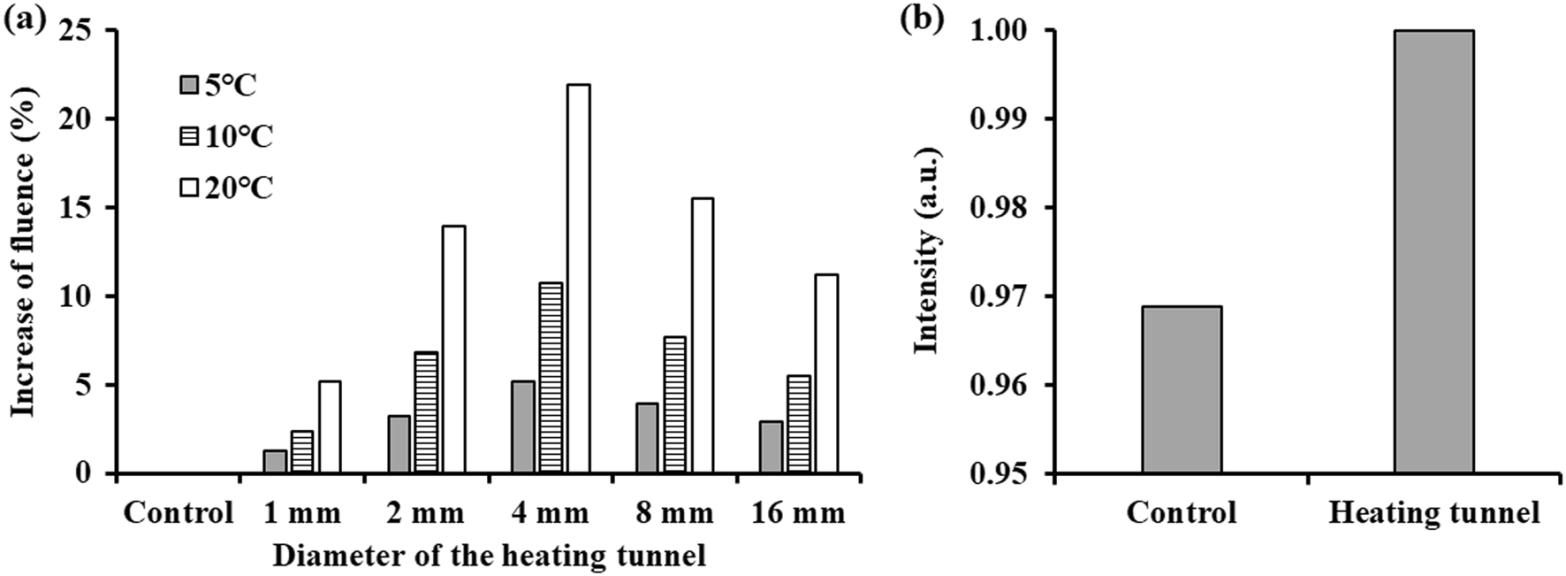
Results from Monte Carlo simulations of (**a**) the increase of fluence propagating through the scattering phantom under different diameters of the heating tunnel (1, 2, 4, 8, 16 mm) and different temperature increase (5, 10, 20 ℃). (**b**) Increase in fluence induced by the four-layer heating tunnel (simulated in the HIFU-sonicated region).

The correlation between fluence improvement, heating tunnel size, and temperature rise are firstly investigated. The result indicates that the increase in the delivered light energy was the greatest when the diameter of the heating tunnel was 4 mm in each temperature (Fig. 3a). Because the photon would be guided by the lower scattering region which was created by the heating tunnel. When the size of the heating tunnel slightly larger than the width of the laser beam, the light fluence would be greatest increased. The light energy could be improved by 5.1%, 10.7%, and 21.9% as temperature increased to 5, 10, and 20 ℃, respectively, showing a positive correlation between light energy improvement and temperature increase. In the end, the simulation results from the actual HIFU heating model are shown in Fig. 3b, which indicate that the HIFU-induced heating increased the light energy by 3.2%.

### Inertial cavitation detection during heating

**Figure 4:**
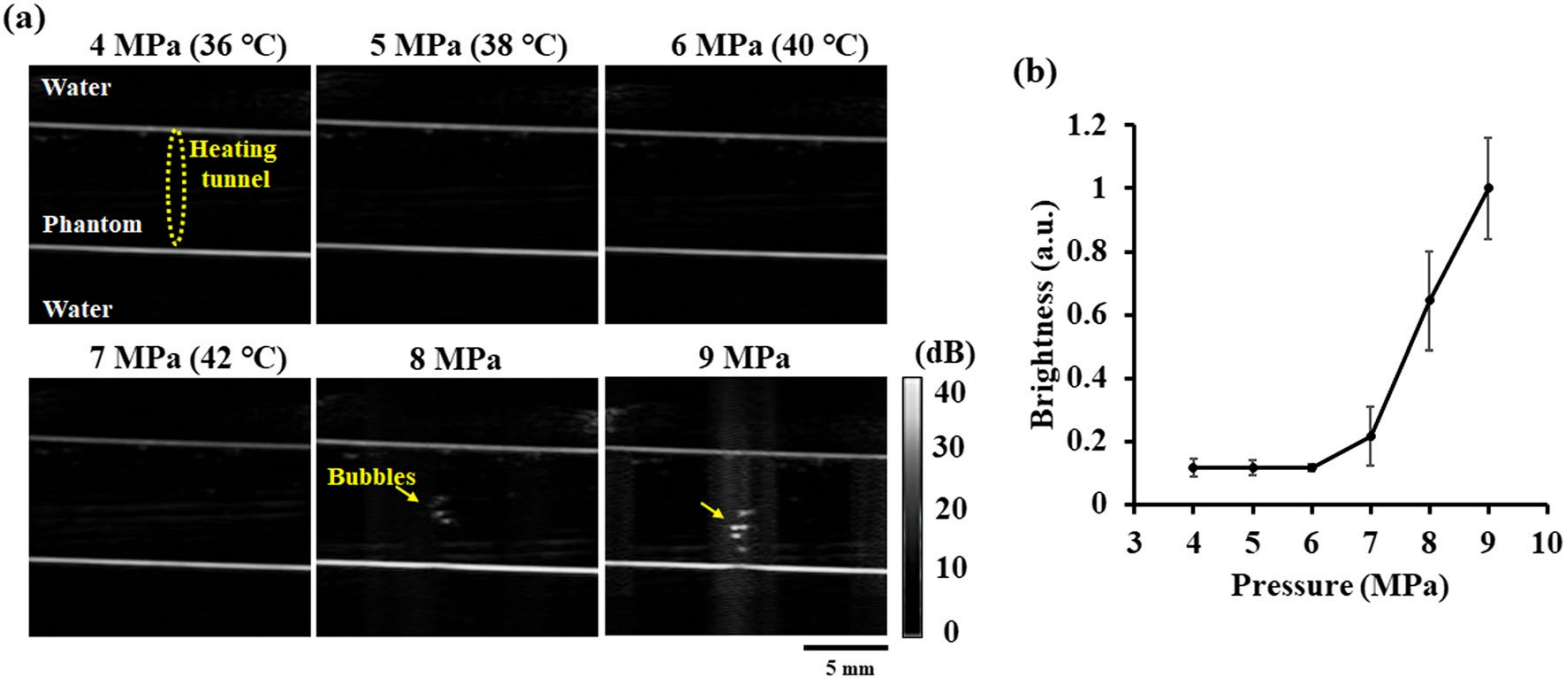
(**a**) The ultrasound B-mode images acquired during HIFU heating with different parameters (4–9 MPa, 100 Hz pulse repetition rate, 7600 cycles, 50% duty cycle, 60 s). (**b**) The backscattering signal intensity within heating area measured from ultrasound B-mode images.

Generating the heating tunnel in the phantom requires the sonication of HIFU at a high acoustic pressure. However, previous studies had indicated that high pressure of ultrasound might induce inertial cavitation^19,20^, producing air bubbles in the sonicated area. These air bubbles also would alter the optical properties of tissue and affect the fluence measurements. In order to verify that the acoustic parameters used in this study will not produce air bubbles, 4–9 MPa acoustic pressure ultrasound signals with 50% duty cycle were used to irradiate the phantom. In the meantime, we used ultrasound B-mode imaging to detect backscattering signals of the generated air bubbles within the phantom. During the experiment, two thermocouples were used to record the temperature change at the center of the heating tunnel and in the water tank. The results show that no air bubble was observed in the B-mode ultrasound imaging until the acoustic pressure exceeded 7 MPa threshold (see Fig. 4). After the pressure was increased to 8 MPa, the air bubble cluster induced by inertial cavitation could be seen in the B-mode imaging. Therefore, we chose 7 MPa pressure as the acoustic parameter for heating to prevent the occurrence of inertial cavitation effect in the phantom.

### Light fluence increased by HIFU-induced heating

**Figure 5:**
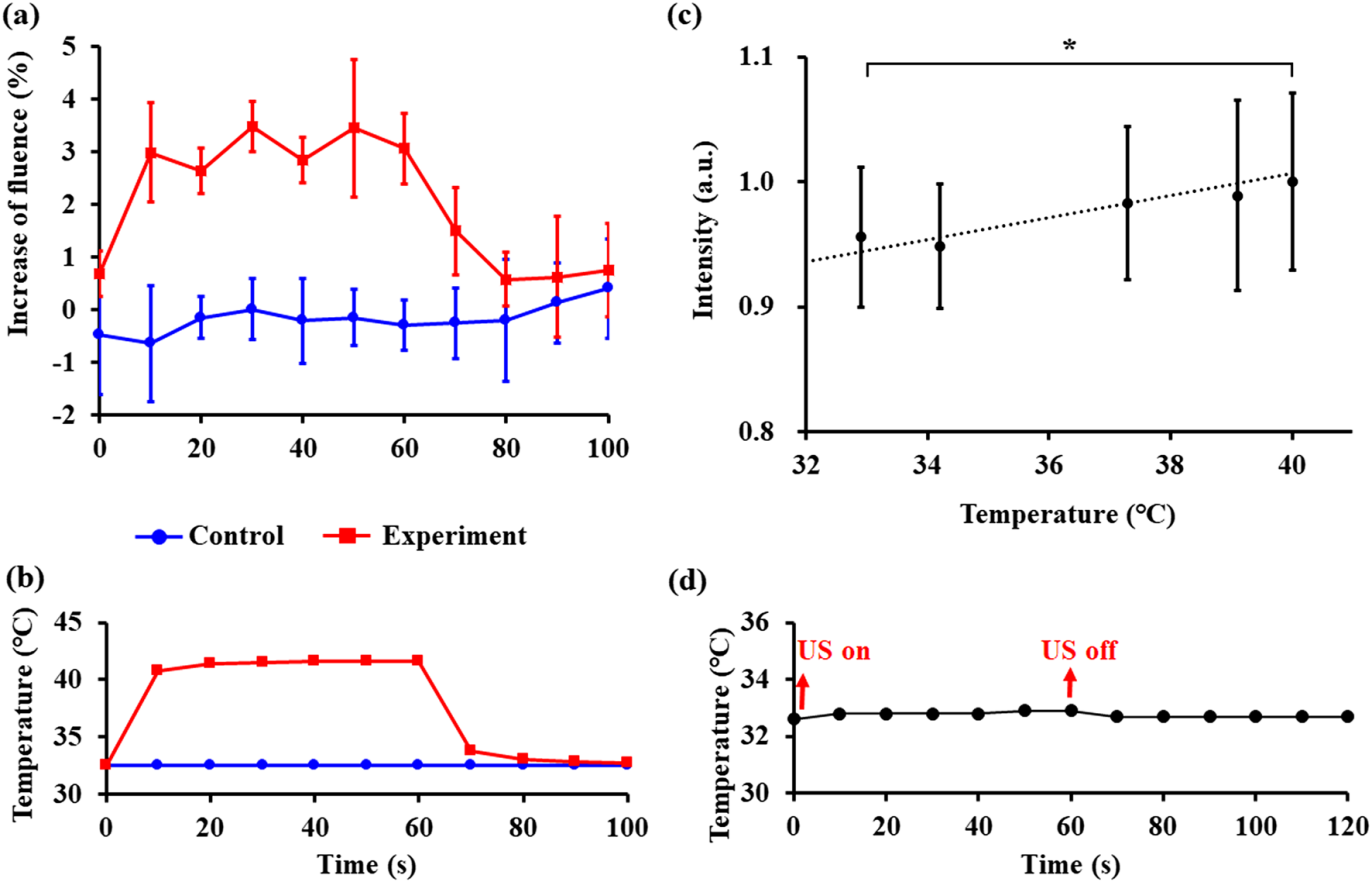
(**a**, **b**) Fluence and temperature increase measured by the photodetector and thermocouple in the in vitro phantom experiment. (**c**) The photoacoustic signal intensity increased with the different temperature of the heating tunnel (*, *p* < 0.05). Note that the temperature of the water bath was almost unchanged due to the special setting of the phantom and an optical absorber (hair) (N = 3 in each experiment). (**d**) The temperature change of the hair during experiment.

The temperatures within the heating tunnel and the laser fluences passing through the intralipid phantom are shown in Fig. 5a,b. The temperature increase in the control group (without heating) and HIFU heating group were highly consistent in each experiment (N = 3) (Fig. 5a). However, the ± 1% standard deviation of the increase of the fluence shown in the control group was caused by the output variation of the laser itself. After turning on the HIFU transducer for 60 s, the light energy was increased by 3.1 ± 0.3%. When the HIFU transducer was turned off after 60 s, the fluence suddenly decreased to the control level, with this decrease synchronized with the temperature change shown. These observations demonstrated the reversible nature of our method.

### Photoacoustic signal enhancement

In the photoacoustic experiment, the intensities of the photoacoustic signals at different temperatures from the heating tunnel were recorded by the oscilloscope. The intensity of the photoacoustic signal increased by 3.0 ± 0.7% solely as a result of the temperature increase of the heating tunnel from 32 to 42 °C (Fig. 5c) because the temperature of the hair did not change (32.7℃) during HIFU heating (see Fig. 5d). The temperature of the water bath was almost unchanged in the HIFU heating process due to the special setting of the phantom and an optical absorber (hair), which demonstrating the fluence increase was only induced by the heating tunnel.

## Discussion and conclusions

The results show that the highly match between the simulation data and in vitro experiment results, confirming the change of the anisotropy factor is neglectable. While we have demonstrated that our proposed strategy based on HIFU-induced heating can rapidly increase the light fluence, the HIFU energy needs to be carefully regulated in order to avoid causing thermal damage and inertial cavitation in the focal area. Tissue temperatures exceeding 42 °C will cause the coagulation of proteins and lipids, while temperatures above 60 °C will induce coagulation necrosis^21^. Moreover, when the ultrasound pressure exceeds the inertial cavitation threshold of 8 MPa, the shock wave induced by the destruction of bubbles may cause bleeding and membrane perforation^22^.

This study was only conducted in an intralipid phantom. The feasibility of applying this technique in other types of biological tissues therefore requires further investigation, since it has been reported previously that the thermal effects induced by changes in light penetration vary with the composition ratio of proteins and lipids in the tissue^17^. A local temperature increase will decrease the scattering coefficient only in a medium that has a negative temperature coefficient, that is, the parameter indicating the relationship between temperature and the scattering coefficient. A negative temperature coefficient is common in lipid-rich tissues. On the other hand, in the protein-rich tissues such as chicken breast, the positive temperature coefficient will increase the scattering coefficient when the temperature rise^16^. Our method is particularly suitable for applications in such lipid-rich tissues, such as the breast.

This research only addressed the localized thermal effect-enhanced light penetration. Therefore, the increasing of the light fluence is only 3%. To further demonstrate feasibility of this strategy, we expanded the change of the temperature (5, 10, 20 ℃) and the diameter of the heating tunnel (1, 2, 4, 8, 16 mm) to investigate the relationship between the fluence improvement and these two factors. The fluence increase could be up to 5.1%, 10.7%, and 21.9% as the temperature increased to 5, 10, and 20 ℃, respectively, according to the simulation result (Fig. 3a). In addition, on the other hands, other effects of ultrasound such as acoustic-pressure and inertial-cavitation-induce air bubbles also have potential in improving the light delivery. One study found that a high-acoustic-pressure standing wave can be used to change the refractive index of the medium so as to create a structure resembling an optical fiber^14^. Another study showed that light scattering can be inhibited by generating an air bubble in the region where light is propagating^20^. Combining these two effects might make it possible to further improve the efficiency of light transmission through a scattering medium. However, the occurrence of inertial cavitation in the tissue would remove the reversible nature of the intervention.

In conclusion, a real-time, noninvasive, and local HIFU heating system has been constructed that can increase the efficiency of delivering light to tissue. Both simulations and photodiode measurements verified an increase of 3% in the fluence resulting from an HIFU-induced reduction in scattering. The fluence increase was also verified in photoacoustic signal measurements. For future biomedical applications, we consider that this method potentially can be used in in vivo two-photon microscopic imaging system. Several papers had successfully integrated the ultrasound transducer onto the two-photon fluorescence microscopy study to observe the ultrasound-induced cerebrovascular dynamics^23,24^. The imaging depth is largely decreased because of the tissue thickness of mice. Therefore, the proposed technique potentially can be used to improve the imaging depth of the currently existing intravital imaging system. Besides, we also consider this method can be utilized in the in vitro high-resolution optical microscopy image for tissue scanning. This imaging technique also suffers low penetration depth because of the exponentially energy decay due to the multiple light scattering^25^. In this case, the temperature change can be extended to 20 ℃ to further increase the penetrated fluence of the light.

## Materials and methods

### Heat-induced reduction of optical scattering coefficients: background and theory

When light propagates through a biological tissue, the strong scattering caused by tissue decreases the delivered light energy. The optical scattering coefficient (*μ*_*s*_) can be expressed as^26^,

1 $$ \mu_{s} = 3.28\pi r^{2} \rho_{s} \left( {\frac{2\pi r}{\lambda }} \right)^{0.37} \left( {\frac{{n_{s} }}{{n_{m} }} - 1} \right)^{2.09} $$

where *r*, *ρ*_*s*_, and *n*_*s*_ are the radius, density, and refractive index of the scattering particles, respectively, and *n*_*m*_ is the refractive index of the medium. According to Eq. (1)**,** the key factors for reducing the scattering coefficient include decreasing the density of the scattering particles and the refractive index mismatch between scattering particles and the medium. Previous studies shown that when lipids within a tissue were heated from 25 to 45 °C, the structure of the lipid changes from the gel phase through a stable crystalline phase to a liquid-crystalline phase^17,27,28^.The increase of fluidity and mobility would lead to the decrease in both the density of the lipid (*ρ*_*s*_) in the tissue and the refractive index mismatch between the lipid (*n*_*s*_) and the surrounding region (*n*_*m*_). Therefore, temperature raise would decrease the scattering coefficient in the tissue.

### Monte Carlo simulation

The feasibility of using a heating tunnel to improve the delivery efficiency of light was first investigated using the MCX Monte Carlo simulation software^29^. Two simulation models were designed in this study (Fig. 1a). The first model was used to specify the different temperature increase and optimize the size of the heating tunnel. A Gaussian-beam laser with a full width at half maximum (FWHM) of 2 mm was selected as an incident light source, and 10^7^ photon packages were launched to simulate the light propagation. The parameters of the tissue-mimicking phantom (intralipid phantom) were initialized as a 20 × 20 × 20 mm^3^ cube with an absorption coefficient (*μ*_*a*_) = 0.05 mm^–1^, anisotropy (*g*) = 0.9. In order to optimize the size of the heating tunnel, the heating tunnel in the phantom was set as a 5-mm-height single layer cylinder with five different diameters. The diameters smaller than (diameter = 1 mm), equal to (diameter = 2 mm), and larger than (diameter = 4, 8, 16 mm) the beam width of the incident light were simulated. The relationship between the fluence improvement and temperature rise (5, 10, and 20 ℃) was investigated by setting the initial temperature of the cube from 37, 32, and 22 ℃ (the scatter coefficient (μs) of the cube = 15.7, 16.6, 18.4 mm-1 followed the experiment results of Cletus^15^). On the other hand, during the HIFU heating process, the actual heating area will not be a homogeneous region and instead will be a multilayer shape with different temperature gradient^30^ and the max temperature increasing can only reach 10 ℃ considering not to induce the inertial cavitation effect in the scattering medium. Therefore, a second model was constructed with a four-layer heating tunnel using the temperature-dependent scattering coefficient (Fig. 1b) to simulate the HIFU-induced thermal effect. In this simulation, the number of photon packages and the phantom parameters were the same as in the first model, while a Gaussian beam laser with an FWHM of 1 mm was chosen to simulate the light spot produced by our 633 nm laser generator. The light fluence in the two simulation models were measured at a depth of 5 mm to observe the light energy variance before and after using heating tunnel. The light fluence was determined by summing the light energy within an area of 1 mm^2^ to simulate the photodiode detection area.

### Inertial cavitation detection during heating

After the simulation, an in vitro phantom experiment was firstly designed to verify the acoustic parameters used in this study will not produce inertial cavitation and thus air bubbles within the phantom during heating (see Fig. 2a). The phantom and the 1.5-MHz HIFU transducer were placed in a homemade acrylic water tank at 32 ℃. An agar-based intralipid phantom was fabricated to simulate the optical scattering and temperature-induced changes in tissue in our experiment. Briefly, 0.9 g of agarose powder (UltraPure Agarose, Invitrogen, Carlsbad, CA, USA), 0.6 ml of intralipid solution (20% Lipovenoes, Fresenius Kabi, Graz, Austria) and 60 ml of distilled and degassed water was infused into a homemade mold (length, 55 mm; width, 35 mm; height, 5 mm), with the intralipid phantom being formed when the agar gel congealed. The heating tunnel within the intralipid phantom was created by using this spherically annulus 1.5-MHz HIFU transducer (H-186, Sonic Concepts, Bothell, WA, USA) driven by a waveform generator (AFG3022C, Tektronix, Beaverton, OR, USA) via a radiofrequency power amplifier (150A100B, Amplifier Research, Hazerswoude-Dorp, Netherlands). To verify the acoustic parameters used in this study will not produce inertial cavitation and air bubbles within the phantom during heating, a 7.5-MHz linear array imaging transducer (model t3000, Terason, Burlington, MA, USA) was placed into the central hole of the 1.5-MHz HIFU transducer to detect backscattering signals of the generated air bubbles via ultrasound B-mode imaging during HIFU heating with different acoustic parameters (i.e., 4–9 MPa, 100 Hz pulse repetition rate, 7600 cycles, 50% duty cycle, 60 s). During the experiment, two thermocouples (HYP-1, Omega Engineering, Stamford, CT, USA) were used to record the temperature change at the center of the heating tunnel and in the water tank. The images obtained at different acoustic pressures were processed with MATLAB software (MathWorks, Natick, MA, USA) to evaluate the backscattering signals in the HIFU-sonicated area.

### Light fluence increased by HIFU-induced heating

The experimental setup for evaluating the correlation between fluence improvement and temperature raise was shown in Fig. 2b. The phantom and HIFU transducer were placed in a homemade acrylic water tank at 32 °C. The heating tunnel in the intralipid phantom was created by the HIFU transducer. A 5-mW, 633-nm, continuous-wave laser (LHR150, Melles Griot, Rochester, NY, USA) was chosen as a light source. The laser head was placed at the center of the HIFU transducer to ensure the light would be effectively transmitting into HIFU-induced heating tunnel. A photodiode (DET10A, Thorlabs, Newton, NJ, USA) with 1 ns reacting time connected to an oscilloscope (TBS 1052B, Tektronix) was placed outside the water tank. An ultrasound waveform with 100 Hz pulse repetition rate and 7600 cycles (50% duty) was applied for 60 s (inducing a pressure of 7 MPa) to generate the 10 °C temperature increase in the phantom. During the experiment, a thermocouple was inserted at the site of the HIFU sonication area to monitor the temperature change within the phantom. The thermocouple confirmed that a 5-mm-long and 1-mm-wide ellipsoid-shaped heating tunnel was created. The transducer was then turned off for 40 s to allow the phantom to cool down. The light fluence as received by the photodiode was displayed on the oscilloscope as a DC (direct current) signal during the heating process. The intensities of received signals were normalized to that acquired before heating in order to evaluate the fluence improvement produced by the HIFU-induced heating. This experiment was independently repeated on three different intralipid phantoms in order to obtain reliable results. Note that the temperature increase was carefully regulated to avoid the occurrence of lipid coagulation, thus the absorption coefficient (*μ*_*a*_) and anisotropy factor (g) were unchanged during the experiment^31^. In other words, this approach only reduced the scattering coefficient (*μ*_*s*_), and then decreased the light scattering in the tissue^15^.

### Photoacoustic experiments

After the in vitro phantom experiment, we chose the photoacoustic system as a common optical device to verify the change in the fluence. The experimental setup is shown in Fig. 2c. A hair—used as an optical absorber—was placed behind the intralipid phantom. This setting is to prevent the photoacoustic signal increasing directly affected by the temperature rise^32^. During the experiment, the thermocouple was attached to the hair for monitoring the temperature change. Because the temperature wasn’t changed during the heating process, the Gruneisen coefficient of theoptical absorber could be regarded as a constant. The laser beam from a 10-mJ, 532-nm Nd:YAG pulsed laser (SLII-10, Continuum Electro-Optics, Milpitas, CA, USA) with 1 kHz pulse repetition rate passing through the heating tunnel created by the HIFU transducer was used to locate the hair. The photoacoustic signal generated by the hair was received by a 25-MHz ultrasound transducer (V324, Olympus, MA, USA), amplified by a low-noise preamplifier (model AU-1114-BNC, Narda-MITEQ, Hauppauge, NY, USA), and sampled by an oscilloscope (LT354, LeCroy, Chestnut Ridge, NY, USA). The intensities of the received signals were then normalized to that acquired before heating to evaluate the fluence improvement produced by the HIFU-induced heating.

### Statistical analysis

All of the described experiments were performed in triplicate. Data are presented as mean ± standard-deviation values. All statistical evaluations were carried out using unpaired two-tailed Student’s *t*-tests. A *p* value of less than 0.05 was accepted as representing a statistically significant difference.
